# Comparison of Immediate Implantation into the Socket with and without Periapical Pathology: Systematic Review and Meta-Analysis

**DOI:** 10.3390/medicina60060893

**Published:** 2024-05-28

**Authors:** Alma Pranckeviciene, Inga Vaitkeviciene, Jolanta Siudikiene, Skaiste Poskeviciene, Vita Maciulskiene-Visockiene

**Affiliations:** Departament of Dental and Oral Pathology, Lithuanian University of Health Sciences, Mickeviciaus 9, 44307 Kaunas, Lithuania; almapran1003@kmu.lt (A.P.); inga.vaitkeviciene@lsmu.lt (I.V.); jolanta.siudikiene@lsmu.lt (J.S.); skaiste.poskeviciene@lsmu.lt (S.P.)

**Keywords:** immediate implantation, immediate implant placement, periapical pathology, periapical lesion

## Abstract

*Background and Objectives*: The present systematic review and meta-analysis were conducted to evaluate and compare the long-term clinical outcomes of immediate implants placed into fresh sockets with and without periapical pathology. *Materials and Methods*: After the search and review of the literature in the electronic databases, 109 publications were achieved. The titles and abstracts of 66 publications were screened. After the evaluation of the full text of 22 publications, based on the inclusion criteria, six controlled clinical studies were included in this systematic review and meta-analysis. *Results:* The statistical calculation showed no heterogeneity among the studies included. The implant survival was 99.6% in the test (socket with periapical pathology) and control (socket without periapical pathology) groups of all the clinical trials. The results of the meta-analysis showed no statistically significant difference between test and control groups regarding the marginal bone level and the width of keratinized mucosa in all the studies. Other parameters indicating plaque level, bleeding on probing, and gingival recession also did not differ between test and control groups at the final follow-up in nearly all studies. *Conclusions*: Within the limitation of this systemic review and meta-analysis, the obtained data suggest that implants immediately placed into the extraction sockets of teeth exhibiting periapical pathology can be successfully osseointegrated for an extended period.

## 1. Introduction

Immediate implant placement after tooth extraction in a fresh post-extraction socket is a dental procedure with high success rates, which leverages the organism’s regenerative potential [1]. This implantation technology aims to preserve the pre-extraction contours of the alveolar process that undergoes resorption and remodeling after tooth loss [2,3]. 

Moreover, numerous advantages of immediate implant placement over non-immediate (early, delayed, and late) implantation have been discussed in the scientific literature.

Thus, the clinical studies demonstrated that this one-step procedure can influence the reduction in crestal bone loss as well as help to maintain the dimensions of the osseous tissues and keep the placed implants at the same angulation as pre-existing teeth [1,4]. Implantation into the fresh socket can reduce the number of required surgical interventions, improve aesthetic outcomes and comfort throughout the healing process, and reduce the treatment period from tooth extraction until definitive prosthesis placement [4,5,6].

However, there is an ongoing debate regarding various local factors that can potentially influence the success of immediate implantation. It has been suggested that immediate dental implant placement could be contraindicated due to risks arising from potential microbial interference with the healing process upon the existence of periapical and periodontal lesions [7].

Retrograde peri-implantitis, characterized by radiolucencies occurring around the most apical part of the osseointegrated implant, has been linked to the presence of previous endodontic pathology [8]. This was supported by a clinical retrospective study, showing that retrograde peri-implantitis was provoked by the scar or granulomatous tissue remaining at the recipient sites, further associated with the endodontic pathology of either the extracted teeth or the neighboring teeth [8]. 

While there are numerous factors that could increase the risk of immediate implant placement failure, some investigations show that peri-radicular infections may not lead to inconveniences linked to immediate implantation if appropriate and thorough cleaning and decontamination of the surgical sites are carried out prior to the procedure [4]. Given the lack of a unified protocol for the preparation of implantation sites, there is no consensus on whether the most used decontamination measures, including antibiotic treatment, mouth rinse with chlorhexidine solutions, or laser irradiation, have a definite effect on the success of immediate implant placement.

Overall, immediate implant placement into fresh post-extraction sockets is still open for scientific debate, especially in clinical situations where periapical pathology is present. Thus, a systematic review of the available literature was performed to analyze the results of different clinical studies.

Therefore, the aim of our systematic review and meta-analysis was to evaluate and compare the long-term clinical outcomes of immediate implants placed into fresh sockets with and without periapical pathology.

## 2. Materials and Methods

### 2.1. Protocol and Registration

The report of this systematic analysis adhered to the Preferred Reporting Items for Systematic Review and Meta-Analyses (PRISMA) statement [9].

This review was registered in the international prospective register of systematic reviews called “PROSPERO” under the number CRD42019135632.

The methods of the analysis as well as the inclusion criteria for the studies were specified in advance and documented in a protocol. 

### 2.2. Focus Question

The following focus question was developed according to the PICO (population, intervention, comparison, and outcome) study design (Table 1): 

### 2.3. Types of Publications and Studies

The search was limited to all human prospective, retrospective, cohort, and case-control clinical studies as well as clinical trials. The meta-analysis included controlled clinical trials, in which immediate implant placement into the sockets with periapical lesions was performed. All studies published up to 13 May 2024 were included.

### 2.4. Information Sources

The literature search was performed in the following databases: MEDLINE via the PubMed database of the US National Library of Medicine, PubMed Central, Google Scholar, ScienceDirect, and the Cochrane Central Register of Controlled Trials (CENTRAL). The electronic search was supplemented by a manual search of the bibliographies of all full-text articles. The reference list of each relevant article was screened to find additional relevant publications.

### 2.5. Search

The combination of Medical Subject Heading search terms (MeSH) and free-text terms included the following:

(“periapical lesion” OR “periapical pathology”) AND (“dental implantation” OR “dental implants” OR “immediate dental implantation” OR “immediate implant placement”).

The choice of keywords was intended to be extensive to collect as much relevant data as possible and to refine the search results without relying on electronic means alone.

### 2.6. Selection of Studies

All titles and abstracts were independently screened by two authors (A.P. and J.S.) based on the inclusion criteria. Any disagreements were resolved by discussion between the reviewers and by consulting two experienced senior reviewers (V.M. and I.V.) when consensus could not be reached. Further, full texts were read to confirm each study’s eligibility based on the inclusion and exclusion criteria stated below. Any disagreements were solved through consensus.

### 2.7. Inclusion Criteria

(a) Clinical trials with humans
(b) Randomized controlled clinical trials where extracted teeth with periapical pathology were replaced with immediate dental implants
(c) Patients in the control group had a healthy periapical area in which immediate implants had been inserted
(d) Reports on clinical and radiographical parameters indicating peri-implant tissue health and osteointegration of the implant
(e) Follow-up period ≥12 months after implant placement and clinical outcomes
(f) Original data of the relevant publications can be received

### 2.8. Exclusion Criteria

- Studies not reporting on the clinical and radiographic treatment outcomes
- In vitro studies, animal studies, letters, editorials, theses, commentaries, PhD theses, consensus statements, reviews, and meta-analyses

### 2.9. Risk of Bias

The quality of all the included studies was assessed (Table 2). The Cochrane Collaboration’s 2-part tool was used [10].

### 2.10. Data Extraction

The data were extracted from the studies in the form of variables. 

**Figure 1:**
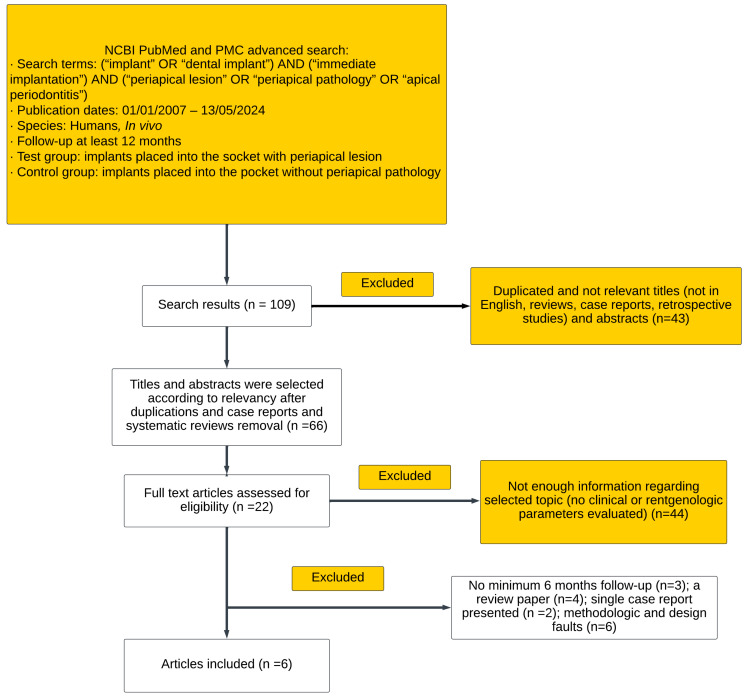
PRISMA flow diagram of the study selection.

The article review and data extraction were performed following the PRISMA guidelines (Figure 1).

### 2.11. Data Items

The data were collected from the included articles and arranged in the following fields:

(a) “Author”—revealed the author and year of publication.
(b) “Study design”—indicated the type of study.
(c) “Baseline records”—revealed the baseline records (clinical and radiological).
(d) “Clinical parameters”—revealed what clinical parameters the authors used for the evaluation of the peri-implant tissues (probing depth (PD), clinical attachment loss (CAL), bleeding on probing (BOP), keratinized mucosa (KM), plaque score (mPI), bleeding on probing (FMPS, BOP, and mBI), and gingival recession (MFR, DPR, and MPR).
(e) “Radiographic evaluation”—described the suggested radiological method to diagnose bone-level changes around the dental implant.
(f) “Number of patients and intervention”—revealed the number of patients treated and the treatment protocols in the test and control groups.
(g) “Number of implants”—revealed the number of implants immediately placed after tooth extraction.
(h) “Follow-up”—revealed the time in months/years during which the patients in the test and control groups were followed.
(i) “Treatment outcomes”—revealed the clinical parameters and their values at the baseline and after a follow-up period (i.e., CAL, PD, and/or BOP) to describe implant failure/success.

The characteristics of the studies included are summarized in Table 3.

### 2.12. Statistical Analysis

The meta-analysis was performed using SPSS statistical software (version 29.0, SPSS Inc., Chicago, IL, USA) and MedCulc. The heterogeneity of the studies was tested using the Cochrane Q test, where I^2^ = 100% × (Q − df)/Q. In the present meta-analysis, Q/df was (2.632/5 = 0.7565) statistically insignificant and showed no heterogeneity. 

## 3. Results

In total, six clinical studies were included in the present meta-analysis. Among them, five were prospective randomized controlled trials [2,3,4,5,7] and one was a prospective cohort study [1]. The data of 271 patients (the reported age ranged from 18 to 87 years) and 251 implants in the test and control groups were collected. In five studies, the teeth extracted due to infection included incisors, canines, and premolars, and in one study, all the tooth groups were included. In four studies [1,2,3,5] the teeth in the test group were extracted due to acute periapical pathology, and in two studies [4,6], the teeth were extracted due to chronic lesions of endodontic origin. A systematic analysis of all the data showed that immediate implant placement in sites with or without periapical pathology did not lead to implant failure during the selected follow-up periods: the survival rate of 99.6% was reported for 250 implants in both the test and control groups. One implant was lost during follow-up due to improper oral hygiene of the patient. The long-term follow-up period ranged from 12 months to 5 years. There were two studies with a follow-up period of 1 year, one with 2 years, two with 3 years, and one with 5 years [1,2,3,4,5,7].

The preparatory principles for the extraction of infected teeth differed among the studies. Antibiotic prophylaxis was performed in four studies [1,2,4,7]. The patients received amoxicillin in two studies [4,7], penicillin in one study [2], and 875/125 mg amoxicillin/clavulanic acid in one study [1]. In the pre-operative period, antibiotic treatment was started 4 days or 1 h before the immediate placement of an implant after tooth extraction [1,2,4,7]. 

All studies reported the preparation of the alveola for immediate implantation. After tooth extraction and thorough removal of all granulation tissues, in three studies, the socket was additionally treated, rinsed with a physiologic solution, disinfected with 0.12% chlorhexidine-soaked gauzes (applied for 1 min), and abundantly irrigated with physiological serum and 0.12% chlorhexidine or cleaned with 90% hydrogen peroxide and irradiated with (Er,Cr:YSGG) laser [1,4,7].

In four studies, guided bone regeneration was performed using deproteinized bovine bone mineral and resorbable collagen or tetrafluoroethylene membrane [2,3,4,5]. Camara and co-workers only used bovine bone substitutes [1]. In all the studies, the patients in the test and control groups were prescribed irrigation with 0.12% or 0.2% chlorhexidine digluconate solution for 4–15 days in the post-operative period. 

The clinical evaluation of all implants was performed in all the clinical trials included in this review. The evaluation of gingival bleeding differed between the studies. Three studies reported data on FMBS, two studies reported data on mBI, and one on BOP [1,2,3,4,5,7]. All studies included data on KM and IS-BIC. The data analysis of the studies indicated that after the long-term evaluation period, no statistically significant differences in the periodontal clinical and radiographical parameters were found between the test and control groups at the final follow-up, except for FMBS in one study [5] and PD in another study [7] (Table 4). 

**Figure 2:**
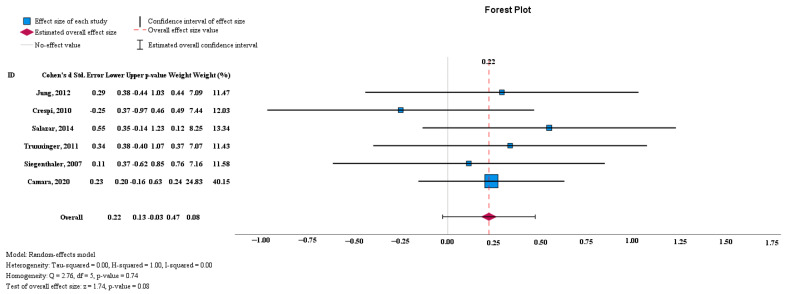
The KM odds ratio and weight for each study [1,2,3,4,5,7].

The results displayed in the forest plot show no significant difference between the mean KM values in the control and test groups (0.22 [95% CI, −0.03 to 0.47], *p* > 0.05). A high level of statistical homogeneity was observed for the meta-analysis of KM (I^2^ = 0%, *p* > 0.05) (Figure 2).

**Figure 3:**
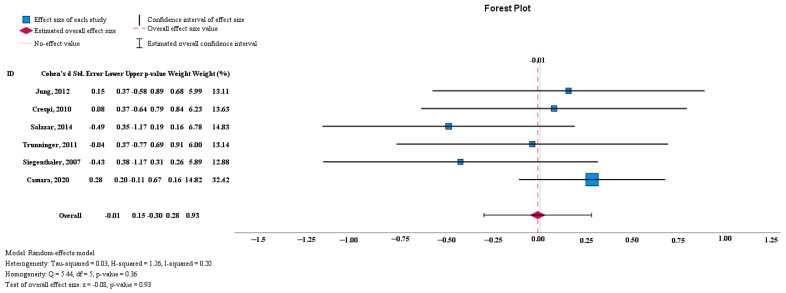
The BIC odds ratio and weight for each study [1,2,3,4,5,7].

The results displayed in the forest plot show no significant difference between the mean BIC values of the control and test groups (−0.01 [95% CI, −0.30 to 0.28], *p* > 0.05). A high level of statistical homogeneity was observed for the meta-analysis of BIC (I^2^ = 0.2%, *p* > 0.05) (Figure 3). 

## 4. Discussion

The key question of this literature review was whether immediate implant placement in humans can provide optimal aesthetic and functional outcomes, even in clinical cases involving periapical lesion presence.

According to the results of the data analysis, there were no significant differences between the control and test groups regarding implant failure; the overall survival rate was 99.6% at the end of the follow-up period. Only one implant was lost during a 3-year follow-up due to the poor oral hygiene of the patient [4]. This finding is in agreement with the results from other studies where the implant survival rate following immediate placement in the infected sockets was 92–100%, with no significant differences compared to the survival rates of immediate implants in the non-infected sockets [6,11,12]. However, it should be noted that in the studies by Trunninger et al. (2011) and Siegenthaler et al. (2007), five patients had to be withdrawn at the early post-implantation stage because primary implant stability could not be achieved [2,3]. 

Implant survival and/or implant success are directly related to the condition of the soft periodontal tissue and the alveolar bone after implantation. This condition can be evaluated using clinical and radiological parameters. All the studies included in our review evaluated and reported the key parameters that help to determine the success of implantation, such as bleeding on probing, keratinized mucosa level, and marginal bone level. Any changes in these parameters may be related to the contamination of implantation sites due to residual infection arising after the extraction of teeth with endodontic infection. Alternatively, the changes could also be linked to insufficient infection control during the implantation procedure or the initial healing period. Thus, there is no definite answer to the question regarding which factors determine the success of immediate implant placement in a previously contaminated area.

Nelson and Thomas (2010) studied whether it was possible for an extra-radicular infection to persist in a healed alveolar bone. The authors concluded that, following a surgical removal of teeth with apical or radicular pathosis, bacteria may linger in the alveolar bone that appears to have healed [13]. Furthermore, it has been suggested that one of the endodontic pathogens linked to peri-implantitis—*E. faecalis*—can persist in the osseous environment after tooth extraction due to failed endodontic treatment. This can further provide surfaces for bacteria to colonize after dental implant placement [14]. 

However, Yu et al. (2015) reported that immediate implant placement in the presence of pre-existing infected periapical sites may not necessarily be contraindicated if appropriate clinical procedures such as antibiotic administration, socket cleaning, and alveolar debridement are performed prior to implantation [15]. Furthermore, the extent of the clinician’s experience in preparing the implantation sites (e.g., in granulation tissue differentiation and debridement as well as in guided bone regeneration procedures) and effective patient cooperation are the key factors to reach a successful outcome [16,17]. 

All studies included in this review and meta-analysis evaluated bleeding on probing by calculating BOP, the FMBS, or the mBI. The amount of bleeding slightly increased throughout the follow-up period; however, bleeding on probing was significantly greater in the test group at the final examination only in the study by Jung et al. (2012) [5]. These favorable results could be attributed to effective periodontal maintenance and patient collaboration. 

MBL is an important parameter when determining the success of implantation [13]. It depends on a number of different factors including implant type, implantation site, technical aspects of the procedure, and individual patient characteristics [18,19,20]. Moreover, in the early period after implant placement, MBL can naturally undergo a non-infective bone remodeling process [21]. During the extended period of observation, Jung and co-workers determined that bone loss around implants mainly took place during the first 12 months and then remained stable for a long period of time [5].

According to Albrektsson and Isidor (1994), implant success is legitimate if there is less than 1.5 mm of bone loss during the first year following functional loading and less than 0.2 mm loss per year going further [22]. This review compared the BIC values reported in the included studies to evaluate the MBL and assess how it differed between the control and test groups. Overall, both groups suffered similarly reasonable bone loss during the observation period, and there were no statistically significant differences. It is important to note that the length of the follow-up period differed between the studies—Camara et al. (2020) and Siegenthaler et al. (2007): 1 year, Crespi et al. (2010): 2 years, Salazar et al. (2014) and Trunninger et al. (2011): 3 years, and Jung et al. (2012): 5 years [1,2,3,4,5,7]. Such differences in study duration should be accounted for when assessing the final BIC values, keeping in mind the criteria previously described by Alberktsson and Isidor [22]. No control groups had BIC values above what Alberktsson and Isidor (1994) considered to be a legitimate bone loss, taking the length of the observational period into consideration. At the end of the respective follow-up periods, more than 1.5 mm of bone loss was found in the test groups in the studies by Jung et al. (2012), Trunninger et al. (2011), and Siegenthaler et al. (2007); however, only Siegenthaler et al. (2007) reported bone loss > 1.5 mm 1 year after implantation [2,3,5]. Additionally, >1.5 mm bone loss was observed only in the distal part of the implantation site. Moreover, no radiolucencies were observed around the tip of the implant in the test groups of all six studies. Overall, the results of the included studies suggest that the presence of infection at the implantation site did not have a significant effect on MBL.

Another important factor that can affect the implantation success is the amount of surrounding soft tissues, primarily the keratinized gingiva. A narrow zone of peri-implant keratinized mucosa (<2 mm) can result in higher BOP and PI levels, gingival recession, and bone loss [23,24]. Keratinized mucosa analysis of the studies revealed that the position of the gingival margin at the implant site and the two neighboring teeth remained stable [1,2,3,4,5,7]. After the follow-up period, soft tissue values were stable, with no recession at the implant sites or the neighboring teeth [1,2,3,4,5,7]. Gingival margin stability was achieved by atraumatic extraction, avoiding raising the full-thickness flap, as well as correct implant positioning [5,7].

The preservation of the crestal bone level is important for implant stability, papilla formation, and infection control associated with the amount of KM. Therefore, guided bone regeneration (GBR) was performed in five of the analyzed studies following the standardized clinical protocols—using deproteinized bovine bone mineral inside and outside the extraction socket and, if needed, a resorbable membrane [1,2,3,4,5,7]. The success of the implantation procedure is determined not only by implant osseointegration but also by the stability of the hard and soft tissues around it [25]. There were no statistically significant differences between the analyzed control and test groups, not only in implant survival but also in KM and IS-BIC values. The implants remained stable and reached optimal aesthetical outcomes. This indicates that immediate implantation is suitable in clinical cases both with or without infection in the post-extraction socket.

Other parameters like mPI, mBI, FMBS, and FMPI, reported in the included studies but excluded from the results of this meta-analysis, remained stable throughout the follow-up period [1,2,3,4,5,7]. 

The surgical protocols of the included studies differed, and there was no standard treatment approach. Granulation tissue found in the infected post-extraction sites of the test groups was debrided in all studies. Thereafter, decontamination of the implantation site with previous infection was performed in two studies using different methods—Camara et al. (2020) irrigated the implantation site with 0.12% chlorhexidine, while Salazar et al. (2014) irrigated it with 90% hydrogen peroxide and used additional laser irradiation [1,4]. Thus, all the investigators recognized that granulation tissue removal was a key factor to ensure successful implantation.

Supplementary antibiotic therapy was applied in all the studies included in the review [1,2,3,4,5,7]. However, it is important to note that the prescription of antibiotics in healthy patients as a preventive strategy to reduce post-operative infections is still debatable [26]. For instance, Hosseini et al. (2015) reported that survival rates of immediate implants in periapical lesions were 100% when using systemically administered antibiotics and 78% without antibiotics [27]. Furthermore, a literature review investigating the efficacy of supplementary antibiotic use before and after implantation showed very similar implantation success rates ranging between 92 and 97% when no antibiotics were used and when prophylactic pre- and post-operative antibiotics were prescribed [28]. 

Our findings support the conclusions presented in the earlier systematic review and meta-analysis [29]. The authors of that review also found no statistically significant differences regarding bone loss and keratinized mucosa level around the implants immediately placed into the sockets with and without periapical pathology. However, in contrast to the present analysis, Kaur et al. (2021) included retrospective split-mouth experimental non-randomized studies, studies without a control group, and studies in which no data regarding the marginal bone level around implants was provided.

One of the limitations of the present systematic review and meta-analysis was that most implants were placed into the region of anterior teeth and premolars. Only Camara et al. (2020) stated that implants were placed in all regions of the dentition [1]. Therefore, there is a need for further controlled clinical studies investigating immediate implant placement in fresh alveolar sockets with periapical pathology, particularly in the posterior regions of the oral cavity. More data available in this field would allow researchers to investigate the potential impact of the implant location in the dentition on the survival rates of implants immediately placed into the socket with periapical pathology.

Another limitation is that only two clinical parameters—KM and IS-BIC—were included in this meta-analysis as all the other important periodontal parameters differed among the selected studies. For instance, bleeding on probing was evaluated by calculating either BOP, FMBS, or mBI. Furthermore, gingival recession was only measured by Salazar et al. (2014) and Camara et al. (2020) [1,4]. Finally, the plaque levels were evaluated in all the studies, except that by Camara et al. (2020), but the indices used were different [1]. However, marginal bone level and the width of keratinized mucosa were the principal parameters indicating the functional and aesthetical stability of the osseointegrated implants. 

Moreover, the surgical protocol and implantation site decontamination methods differed among the studies. Nevertheless, granulation tissue debridement and supplementary antibiotic therapy were included in all the clinical trials discussed here. All of them reported the beneficial outcomes, irrespective of the chosen antibiotic and decontamination method. This indicates that immediate implant placement into the socket with periapical pathology should be performed only after thorough elimination of the infected tissues followed by socket disinfection and should be supported with antibiotic therapy. 

The fact that the periapical lesions were not uniform among the studies limits the generalizability of the findings as well. Chronic pathology was determined in two studies [4,6], while the other four studies indicated an active course of the periapical lesions of the teeth referred for extraction [1,2,3,5]. However, the patients with active periapical lesions received a pre-operative course of antibiotics and rinsing with 0.12% chlorhexidine digluconate, which could mitigate the symptoms and restrain the inflammation. Nonetheless, the potential impact of the periapical inflammation stage on the final outcomes cannot be excluded and needs further investigation. 

Other variations between the studies included the duration of the follow-up period (from 1 to 5 years) and the number of implants placed into fresh sockets with periapical lesions (from 13 to 50). It is important to mention that a minimum of one year of follow-up is required as the greatest changes in the bone level around the implants occur within the first year after implantation. Moreover, the bone resorption levels can be confirmed only in about 2 years following the implant loading. Therefore, more studies with extended follow-up periods are desirable to support the conclusions based on the present data [5,20]. 

## 5. Conclusions

Within the limitations of this systemic review and meta-analysis, the obtained data suggest that implants immediately placed into the extraction sockets of teeth exhibiting periapical pathology can be successfully osseointegrated for an extended period provided the infection control, particularly when thorough debridement of the granulation tissues and decontamination procedures are performed. 

## Figures and Tables

**Table 1:** PICO study design.

Component	Description
Population (P)	Patients in need of the immediate implant retained restoration
Intervention (I)	Immediate implant placement after the extraction of the tooth with periapical pathology
Comparison (C)	Immediate implant placement after the extraction of the tooth without periapical pathology
Outcome (O)	Marginal bone and keratinized mucosa level around implants immediately placed into the socket with and without periapical pathology
Study Design	Controlled randomized clinical trials and prospective cohort studies
Focus question	What is the effect of immediate implantation on the alveolar bone margin and soft tissue dimensions in patients exhibiting periapical lesions and needing an implant retained restoration?

**Table 2:** Risk of Bias evaluation.

Study	Random Sequence Generation	Allocation Concealment	Blinding of Outcome Assessment	Incomplete Outcome Data	Selective Reporting	Other Sources of Bias
Jung et al. (2012) [5]	?	?	+	+	+	+
Crespi et al. (2010) [7]	+	?	+	+	+	+
Montoya-Salazar et al. (2014) [4]	?	?	+	+	+	+
Trunninger et al. (2011) [3]	?	?	+	+	+	+
Siegenthaler et al. (2007) [2]	?	?	+	+	+	+
Camara et al. (2020) [1]	?	+	?	+	+	+

“+” low risk of bias; and “?” unclear.

**Table 3:** Characteristics of the included studies.

Study	Participants Test/Control	Number of Implants Placed Test/Control	Age of Participants (Years) Range and Mean (SD) Test/Control	Teeth Replaced with Immediate Implants	Clinical Parameters	Radiographical Parameters	Follow-Up
Jung et al. (2012) [5]	12/15	13/16	Test: Range: 31–87 Mean: 53 (not specified) Control: Range: 28–82 Mean: 60 (not specified)	ICP	FMBS FMPS CAL KM	IS-BIC periapical area	5 years
Crespi et al. (2010) [7]	30/15	30 in total	All participants: Range: 34–71 Mean: 52.2 (not specified)	ICP	PD mPI mBI KM MGL	IS-BIC	12 and 24 months
Salazar et al. (2014) [4]	18/18	22/14	All participants: Range: 18–50	ICP	PD mPI mBI KM MGL	IS-BIC marginal	12, 24, and 36 months
Trunninger et al. (2011) [3]	16/13	13/16	Test: Range: 23–82 Mean: 45 (15) Control: Range: 23–77 Mean: 55 (15)	ICP	FMBS FMPS CAL KM	IS-BIC	3 years
Siegenthaler et al. (2007) [2]	17/17	13/16	Test: Range: 23–82 Mean: 45 (15) Control: Range: 23–77 Mean: 55 (15)	ICP	FMBS FMPS KM PAL	IS-BIC	1 year
Camara et al. (2020) [1]	50/50	50/50	Test: Mean: 48.78 (11.18) Control: 47.60 (13.16)	All tooth groups	BOP KM MPR DPR MFR PD	IS-BIC marginal	1 year

ICP: incisors, canines, and premolars; FMBS: full-mouth bleeding score; FMPS: full-mouth plaque score; CAL: clinical attachment loss; KM: keratinized mucosa level; PD: probing depth; mPI: modified plaque index; mBI: modified bleeding index; MGL: the distance between the platform of the implant and the marginal gingiva level; PAL: periodontal attachment loss; BOP: bleeding on probing; MPR: mesial papillary recession; DPR: distal papillary recession; MFR: midfacial recession; and IS-BIC: vertical distance from the implant shoulder to the first bone-to-implant contact mesial/distal.

**Table 4:** Periodontal clinical and radiographical parameters at the baseline and final follow-up.

Study	Clinical Parameters Mean (SD): mm and %		Radiographical Parameters (BIC) Mean (SD): mm	
	Test	Control	Test	Control
	Baseline and Follow-up	Baseline and Follow-up	Baseline and Follow-up	Baseline and Follow-up
Jung et al. (2012) [5]	FMBS Not presented 18.5 (11.2) FMPS Not presented 22.9 (15.7) CAL Not presented 2.8 (1.0) KM Not presented 3.7 (1.2)	FMBS Not presented 9.9 (5.1) * FMPS Not presented 17.5 (12.8) CAL Not presented 3.5 (1.2) KM Not presented 3.3 (1.5)	Mesial surface not presented 1.2 (0.7) Distal surface not presented 1.8 (1.2)	Mesial surface not presented 1.5 (0.7) Distal surface not presented 1.2 (0.9)
Crespi et al. (2010) [7]	mPI 0.49 (0.19) 0.69 (0.29) mBI 0.46 (0.23) 0.72 (0.36) MGL 0.15 (0.08) 0.2 (0.13) KM 3.64 (0.68) Remained stable PD 1.80 (0.64) 1.99 (0.57)	mPI 0.53 (0.2) 0.74 (0.29) mBI 0.49 (0.29) 0.77 (0.33) MGL 0.18 (0.11) 0.25 (0.18) KM 3.83 (0.81) Remained stable PD 1.46 (0.48) 2.05 (0.66) *	1.02 (0.33) 0.86 (0.54)	0.99 (0.27) 0.82 (0.52)
Salazar et al. (2014) [4]	PD 2.46 (0.44) 2.51 (0.44) mPI 1.11 (0.67) 0.88 (0.83) mBI 0.88 (0.58) 0.94 (0.73) MGL 0.88 (0.58) 1.00 (0.59) KM 3.55 (0.92) 3.38 (0.6)	PD 2.39 (0.40) 2.53 (0.44) mPI 0.94 (1.10) 1.00 (1.02) mBI 0.66 (0.76) 1.00 (1.02) MGL 1.16 (0.29) 1.16 (0.24) KM 2.61(0.69) 2.88(1.27)	1.36 (0.39) 0.53 (0.13)	1.16 (0.27) 0.6 (0.16)
Trunninger et al. (2011) [3]	FMBS Not presented 11 (7.0) FMPS Not presented 21 (18.0) CAL Not presented 2.7 (1.0) KM Not presented 3.5 (1.7)	FMBS Not presented 12 (9.0) FMPS Not presented 14 (6.0) CAL Not presented 3.4 (1.3) KM Not presented 3 (1.3)	Mesial surface not presented 1.54 (0.88) Distal surface not presented 1.69 (0.92)	Mesial surface not presented 1.57 (0.57) Distal surface not presented 1.59 (0.8)
Siegenthaler et al. (2007) [2]	FMBS 12 (8) 12 (5.0) FMPS 23 (15) 22 (14) PALb 3.1 (0.66) 3.3 (0.6) KM 5.4 (1.4) 3.2(1.6) PALl 3 (0.4) 2.6 (0.6)	FMBS 10 (5) 14 (0.6) FMPS 15 (6) 21 (13) PALb 2.6 (0.77) 3 (1.3) KM 4.3 (1.5) 3 (1.9) PALl 3 (0.7) 3.1 (1.1)	Mesial surface not presented 1.2 (0.7) Distal surface not presented 1.8 (1.2)	Mesial surface not presented 1.5 (0.7) Distal surface not presented 1.2 (0.9)
Camara et al. (2020) [1]	BOP Not presented 24.99 (4.99) KM Not presented 3.76 (0.62) PD Not presented 3.16 (0.41)	BOP Not presented 11.52 (3.04) KM Not presented 3.5 (1.44) PD Not presented 3.31 (0.94)	Not presented 0.35 (0.51)	Not presented 0.15 (0.87)

FMBS (%): full-mouth bleeding score; FMPS (%): full-mouth plaque score; CAL (mm): clinical attachment loss; KM (mm): keratinized mucosa level; PD (mm): probing depth; mPI: modified plaque index; mBI: bleeding index; MGL (mm): distance between the platform of the implant and the marginal gingiva level; PALb (mm): buccal periodontal attachment loss; PALl (mm): lingual periodontal attachment loss; BOP (%): bleeding on probing; and IS-BIC (mm): vertical distance from the implant shoulder to the first bone-to-implant contact mesial/distal. * significant difference: *p* < 0.05 between test and control groups.

## Data Availability

The data presented in this study can be retrieved upon request, due to privacy reasons.
